# Audiotapes and letters to patients: the practice and views of oncologists, surgeons and general practitioners

**DOI:** 10.1038/sj.bjc.6690284

**Published:** 1999-04

**Authors:** D McConnell, P N Butow, M H N Tattersall

**Affiliations:** Medical Psychology Unit and Department of Cancer Medicine, University of Sydney, Sydney, 2006, Australia

**Keywords:** Dr-pt communication, information provision, audiotapes, patient letters

## Abstract

A range of measures have been proposed to enhance the provision of information to cancer patients and randomized controlled trials have demonstrated their impact on patient satisfaction and recall. The current study explored the practice and views of oncologists, surgeons and general practitioners (GPs) with regards to providing patients with consultation audiotapes and summary letters. In stage 1, 28 semi-structured interviews with doctors were conducted to provide qualitative data on which to base a questionnaire. In stage 2, 113 medical oncologists, 43 radiation oncologists, 55 surgeons and 108 GPs completed questionnaires. Only one-third of doctors had ever provided patients with a copy of the letter written to the oncologist or referring doctor, and one-quarter had provided a summary letter or tape. The majority of doctors were opposed to such measures; however, a substantial minority were in favour of providing a letter or tape under certain conditions. More surgeons and GPs (> two-thirds) were opposed to specialists providing a consultation audiotape than oncologists (one-third). Gender, years of experience and attitude to patient involvement in decision-making were predictive of doctors' attitudes. The majority of doctors remain opposed to offering patients personalized information aids. However, practice and perspectives appear to be changing. © 1999 Cancer Research Campaign


*British Journal of Cancer* (1999) **79**(11/12), 1782–1788
© 1999 Cancer Research Campaign
Article no. bjoc.1998.0284

### Audiotapes and letters to patients: the practice and views of oncologists, surgeons and general practitioners

**D McConnell[1], PN Butow[1] and MHN Tattersall[2]**

[1]Medical Psychology Unit and [2]Department of Cancer Medicine, University of Sydney, Sydney 2006, Australia

**Summary** A range of measures have been proposed to enhance the provision of information to cancer patients and randomized controlled trials have demonstrated their impact on patient satisfaction and recall. The current study explored the practice and views of oncologists, surgeons and general practitioners (GPs) with regards to providing patients with consultation audiotapes and summary letters. In stage 1, 28 semi-structured interviews with doctors were conducted to provide qualitative data on which to base a questionnaire. In stage 2, 113 medical oncologists, 43 radiation oncologists, 55 surgeons and 108 GPs completed questionnaires. Only one-third of doctors had ever provided patients with a copy of the letter written to the oncologist or referring doctor, and one-quarter had provided a summary letter or tape. The majority of doctors were opposed to such measures; however, a substantial minority were in favour of providing a letter or tape under certain conditions. More surgeons and GPs (> two-thirds) were opposed to specialists providing a consultation audiotape than oncologists (one-third). Gender, years of experience and attitude to patient involvement in decision-making were predictive of doctors' attitudes. The majority of doctors remain opposed to offering patients personalized information aids. However, practice and perspectives appear to be changing.

**Keywords:** Dr-pt communication; information provision; audiotapes; patient letters

An increasing majority of cancer patients in Western countries wish to be fully informed, and modern medical ethics emphasizes their right to be so (Goldberg, 1984; Beisecker and Beisecker, 1990; Wiggers et al, 1990; Lantos, 1993). Moreover, a large number of studies now suggest that optimal care for the majority of patients hinges, at least in part, on the provision of adequate information (Derdiarian, 1987; Hack et al 1994). One recent study of 165 adult patients with Hodgkin's disease (Turner et al, 1996) found that 48% of patients were dissatisfied with the amount of information they received at the medical consultation, supporting the contention that doctors sometimes underestimate, or fail to satisfy, patients' desire for information (Blanchard et al, 1988; Wiggers et al, 1990; Butow et al, 1997).

Many cancer patients demonstrate poor recall, and poor understanding of information regarding diagnosis, treatment and prognosis (Mackillop et al, 1988; Simminoff et al, 1989). Dunn et al (1993) found that a sample of 142 cancer patients recalled an average of only 25% of the facts presented in an initial consultation with a medical oncologist, and only 45% of the 'key points' as determined by the oncologist. Poor understanding and recall have been attributed to a range of factors, including patient shock and anxiety (Ley and Spelman, 1965), patient denial (Cassileth et al, 1980), and poor communication techniques and time constraints in the consultation (Ley, 1988).

A range of interventions has been proposed to enhance the provision of accurate and adequate information to patients. Such interventions include training doctors in communication techniques (Lancet, 1995), encouraging patients to attend the consultation with a family member or friend (Fallowfield, 1993) and providing patients with any or all of the following aids: a question prompt sheet (Butow et al, 1994); generalized information booklets and tapes; a consultation summary letter (Damian and Tattersall, 1991) and/or an audio tape recording of the cancer consultation (Dunn et al, 1993). The majority of studies have focused on the provision of summary letters and audiotape recordings.

A large majority of cancer patients are in favour of receiving summary letters or consultation audiotapes (Deutsch, 1992; Tattersall et al, 1994; McHugh et al, 1995). Moreover, in randomized controlled trials, the provision of either a letter or tape has been shown to increase patients' overall satisfaction with their medical consultation (Damian and Tattersall, 1991; Dunn et al, 1993), recall and understanding (Hogbin and Fallowfield, 1989; McHugh et al, 1995) and patient activity in the consultation. Ford et al (1995) found that patients with cancer who received an audiotaped copy of their first interview with an oncologist were more likely to ask for clarification of a previously discussed topic in their second linked interview. Even among patients receiving bad news, satisfaction is reported to be high (Hogbin and Fallowfield, 1989; Tattersall et al, 1994), although McHugh et al (1995) found that poor prognosis patients faired worse in psychological terms than those with a good prognosis after being encouraged to listen to audiotapes of their bad news consultations.

Hack et al (1994) found, in a sample of 35 women with stage 1 or 2 breast cancer, that patients preferred a written copy of their diagnosis over a taped copy. However, Tattersall et al (1994) examined the preferences of 182 cancer patients for several interventions, including letters and tapes, and found that 82% ranked the audiotape as their first option. The audio tape was also ranked above a

*Received 27 May 1998*
*Revised 7 August 1998*
*Accepted 20 August 1998*

*Correspondence to:* P Butow, Medical Psychology Unit, Royal Prince Alfred Hospital, Camperdown, NSW 2050, Australia phone call from the oncologist, a copy of the letter from the oncologist to their doctor, or a talk with an oncology nurse specialist either in person or over the phone. Patients felt that the audiotape was more effective in reminding them of what the doctor said, and was more personal, reassuring and human than the letter.

Qualitative data from several studies suggest that the audiotape and letter perform several other functions. Firstly, patients often share the letter or audiotape with family members, friends and their general practitioner (GP), thus sparing the patient from having to recount the information repeatedly. Secondly, letters and tapes provide patients with the opportunity to go over the information presented in the consultation, permitting recollection and clarification. Thirdly, some patients also find the letter and tape useful as a record; they can file it and know they can refer to it again in the future.

Despite these positive reports, anecdotal evidence suggests that few oncologists or surgeons have incorporated either of these interventions into their practice (Tattersall et al, 1997). To date, only one study has examined the reasons for this discrepancy between research findings and clinical practice (Stockler et al, 1993). This study examined the views of 160 doctors involved in the care of patients referred to one oncologist. A majority of doctors (61%) were in favour of oncologists providing patients with an audiotape recording of their consultation. General practitioners were more in favour of the audiotape than specialists. In total, 36% of doctors felt that there were risks in giving patients such a record, though only 13% felt these were prohibitive. When asked to indicate their preference, 71% of doctors felt that an individualized letter would be better than an audiotape, and 53% felt that patients would prefer a letter.

Notably, these views were restricted to information aids provided by the medical oncologist. The views of other medical and radiation oncologists and surgeons as the potential providers of tapes and/or letters were not investigated and remain unknown.

This exploratory study set out to address this gap in knowledge. Our objectives were as follows:

- To identify the proportion of oncologists and surgeons who provide cancer patients with consultation audiotapes and/or letters.
- To identify the proportion of oncologists, surgeons and GPs in favour of offering cancer patients an audiotape of the consultation and/or a summary letter.
- To identify, and make explicit, the rationale underlying perspectives in favour of, and against, offering patients each intervention.
- To identify other interventions used/preferred by medical and radiation oncologists, surgeons and GPs to address patients' information needs.

### MATERIALS AND METHODS

### Stage 1: qualitative phase and questionnaire development

In Stage 1, three medical and three radiation oncologists were invited to participate in an interview and to provide contact details of their last four patients, and the patients, referring doctors and general practitioners. An invitation to participate was then sent to these patients and their doctors. The potential sample was limited as several patients had been referred by a common doctor.

**Table 1** Sample characteristics

| Characteristics | Oncologists | Surgeons | GPs |
|---|---|---|---|
| Sample size | *n* = 156 | *n* = 55 | *n* = 108 |
| Gender | | | |
| Male | 133 (85%) | 54 (98%) | 65 (60%) |
| Female | 23 (15%) | 1 (2%) | 43 (40%) |
| Years experience | | | |
| Mean | 12.56 | 19.74 | 16.59 |
| Range (s.d.) | 0–39 (8.16) | 4–40 (10.02) | 2–50 (11.33) |
| Speciality | | | |
| Radiation oncologist | *n* = 43 | | |
| Medical oncologist | *n* = 113 | | |
| General surgeon | | 32 (57%) | |
| Other surgeon | | 23 (43%) | |
| Average no. cancer patients per years | Data not collected | Data not collected | |
| < 1 | | | 2 (2%) |
| 1–5 | | | 33 (31%) |
| 6–10 | | | 27 (25%) |
| > 10 | | | 45 (42%) |
| Missing data | | | 1 |

A total of 28 semi-structured interviews with doctors were conducted, including seven with oncologists from three Sydney hospitals (one oncologist was a referring doctor), ten with surgeons and 11 with GPs practicing in the Sydney Metropolitan area. The interviews explored the doctors' views on referral communications (these data have been separately submitted for publication) and on providing patients with consultation audiotapes and letters. All interviews were audiotaped, transcribed and analysed using the constant–comparative method (Glaser and Strauss, 1967).

This analysis provided a basis for the development of questionnaires for each group of doctors. The questionnaires used a Likert scale format to quantitate doctors' practice and views concerning the provision of audiotapes and letters to patients. Open-ended questions elicited the rationale behind doctors' views and preferences for alternative communication strategies. Oncologists and surgeons received an identical set of items; general practitioners were asked only about their views concerning specialists' provision of tapes and letters, and not about their own practice in this regard. Data about gender, specialty, years of experience, number of cancer patients seen per year and views about patient involvement in decision-making, were also collected. The last item was a variant of the Sutherland et al (1989) scale which measures patients' preferred level of involvement in decision-making (five categories ranging from 'patient only' to 'doctor only' making decisions). The questionnaires were piloted with three GPs, surgeons and oncologists to ensure clarity in wording and format.

### Stage 2: quantitative data collection

In Stage 2, medical and radiation oncologists, surgeons and GPs were surveyed. The questionnaire was sent to all surgeons (*n* = 84) and radiation oncologists (*n* = 56) who are members of the Clinical Oncological Society of Australia and to all members of the Medical Oncology Group of the Royal Australian College of Physicians (*n* = 148). The sample of GPs was drawn from the

© Cancer Research Campaign 1999

**Table 2** Strategies to meet patients' information needs: the practice of oncologists ($n$ = 154)

| In what proportion of cases do you *offer* patients: | In all/most cases (%) | In some cases (%) | In no cases (%) |
|---|---|---|---|
| a copy of the letter written to the referring doctor/GP? | 1.3 | 25.2 | 73.5 |
| an individualized summary letter after the consultation? | 2.5 | 20.5 | 76.9 |
| an audiotaped recording of the consultation? | 3.2 | 17.9 | 78.8 |
| general information booklets/tapes? | 78.2 | 19.9 | 1.9 |

**Table 3** Strategies to meet patients' information needs: the practice of surgeons ($n$ = 55)

| In what proportion of cases do you *offer* patients: | In all/most cases (%) | In some cases (%) | In no cases (%) |
|---|---|---|---|
| a copy of the letter written to the oncologist/GP | 3.8 | 32.1 | 64.2 |
| an individualized summary letter after the consultation | 3.8 | 22.6 | 73.6 |
| an audiotaped recording of the consultation | 3.8 | 7.5 | 88.7 |
| general information booklets | 69.8 | 22.6 | 7.5 |

Directory of Members of The Royal Australian College of General Practitioners. A sample of 200 GPs was randomly selected using a randomized block design to ensure a representative proportion from each State and Territory.

### *Statistics*

Descriptive statistics were computed to identify the proportion of doctors who practised and favoured the provision of consultation audiotapes and/or letters to patients. Chi square and Student's $t$-test analyses were used to compare responses of doctors who differed in characteristics such as speciality and gender. Qualitative data in response to open-ended questions were analysed using the constant–comparative method, as for the interview data.

### RESULTS

In total, 113 medical oncologists, 43 radiation oncologists, 55 surgeons and 108 GPs returned completed questionnaires, representing a 76%, 77%, 65% and 54% response rate respectively. Demographic characteristics of the sample are presented in Table 1.

### The practice of oncologists and surgeons

Oncologists and surgeons were asked to indicate the proportion of cases in which they offer patients

1. a copy of the letter written to the referring doctor/GP or oncologist (surgeons only)
2. an individualized summary letter of the consultation
3. an audiotape recording of the consultation
4. general information booklets/tapes.

Results are presented in Tables 2 and 3. These findings suggest that oncologists and surgeons rarely offer patients either an audiotape or letter. Notably, both groups of doctors are more likely to offer patients a copy of the letter they write to other doctors than to provide an audiotape. Offering patients general information booklets is clearly the most common practice, and a consultation audiotape the least common practice for both oncologists and surgeons.

The following doctor characteristics were examined in relation to offering patients communication aids: speciality, gender, years of experience and views on involvement of patients in decision-making. A significantly larger percentage of surgeons (32%) than oncologists (16%) sometimes dictated letters to other doctors in front of the patient ($\chi^2 = 6.3$, $P < 0.01$). There were no differences in practice between medical and radiation oncologists. Surgeons who sometimes dictated letters to other doctors in front of the patient had significantly fewer years of experience (14 years) than those who never did this (22 years), $t_{49} = 2.54$, $P < 0.01$). However, years of experience was not associated with the provision of a copy of this letter, or a personalized letter. A significantly larger percentage of surgeons who sometimes offered patients a personalized letter favoured a collaborative approach to treatment decision-making (41%) versus dominance by either the doctor or patient (0%) ($\chi^2 = 10.6$, $P < 0.001$). As only six surgeons ever offered patients an audiotape of their consultation, there was insufficient variability to analyse predictors of this behaviour. There were no significant associations between the predictors and oncologist behaviour with regard to information aids.

### The views of oncologists, surgeons and GPs

Oncologists, surgeons and GPs were asked to indicate whether they think specialists '*should* offer patients'

1. a copy of the letter written to the referring doctor/GP
2. an individualized summary letter
3. an audiotape recording of the consultation.

The results are presented in Tables 4–6. The results indicate that the majority of doctors in all groups are opposed to specialists offering patients either letter or tape. However, a substantial minority of doctors in each group are in favour of providing either

 © Cancer Research Campaign 1999

**Table 4** Strategies to meet patients' information needs: the views of oncologists ($n$ = 156)

| **Should specialists *offer* patients:** | **Yes (%)** | **No (%)** | **It depends (%)** |
|---|---|---|---|
| a copy of the letter they write to the referring doctor/GP? | 6.4 | 53.8 | 39.7 |
| an individualized summary letter after the consultation? | 18.7 | 45.8 | 35.5 |
| an audiotape recording of their consultation? | 24.5 | 38.7 | 36.8 |

**Table 5** Strategies to meet patients' information needs: the views of surgeons ($n$ = 55)

| **Should specialists (including surgeons) *offer* patients:** | **Yes (%)** | **No (%)** | **It depends (%)** |
|---|---|---|---|
| a copy of the letter they write to the referring doctor or oncologist? | 9.4 | 49.1 | 41.5 |
| an individualized summary letter after the consultation? | 18.9 | 43.4 | 37.7 |
| an audiotaped recording of their consultation? | 9.4 | 66 | 24.5 |

**Table 6** Strategies to meet patients' information needs: the views of GPs ($n$ = 108)

| **Should specialists *offer* patients:** | **Yes (%)** | **No (%)** | **It depends (%)** |
|---|---|---|---|
| a copy of the letter they write to the referring doctors/GP? | 13.2 | 35.8 | 50.9 |
| an individualized summary letter after the consultation? | 48.6 | 18.1 | 33.3 |
| an audiotaped recording of their consultation? | 9.4 | 72.6 | 17.9 |

of the letters or tape under certain conditions (outlined below). A notable finding is the opposition of a clear majority (> two-thirds) of surgeons and GPs to specialists offering patients an audiotape recording of their consultation. In contrast, only 38.7% of oncologists were opposed and a quarter favoured provision of an audiotape (although only 3.2% actually offered them in all or most cases (Table 2)). The most popular option for GPs, and to a lesser extent surgeons, was the individualized summary letter. The individualized letter was favoured by 48% of GPs and a further 33% supported this approach under certain circumstances.

Differences between specialists in attitudes to providing information aids were explored; there were significant differences. More GPs than surgeons or oncologists were in favour of providing patients, in at least some cases, with a copy of the letter written to the referring doctor ($\chi^2 = 8.3$, $P < 0.05$) or a personalized letter ($\chi^2 = 22.3$, $P < 0.00001$). However, many more oncologists than GPs or surgeons were in favour of an audiotape ($\chi^2 = 32.5$, $P < 0.00001$). There were no significant differences in attitudes between medical and radiation oncologists. Surgeons who were in favour of providing patients with an audiotape of the consultation had fewer years of experience (16 years) than those against this practice (22 years), ($t_{49} = 1.9$, $P = 0.06$). There was a similar trend for oncologists in favour of audiotapes to have fewer years of experience (12 versus 14; $P = 0.09$).

A significantly larger percentage of surgeons who favoured provision of a personalized letter to the patient endorsed a collaborative, or patient-dominated, approach to decision-making (97%) than those against this practice (56%) ($\chi^2 = 12.9$, $P < 0.001$). There was a similar trend for oncologists favouring provision of an audiotape to endorse a collaborative, or patient-dominated, approach to decision-making ($P = 0.09$).

Finally, significantly more female (77%) than male GPs (56%) favoured providing a copy of the letter sent to the referring doctor to the patient ($P < 0.05$). Similarly, more female (44%) than male GPs (16%) favoured provision of an audiotape of the consultation ($P < 0.001$). Gender was not predictive of GP attitudes to the individualized summary letter, or of oncologist attitudes towards any of the communication strategies.

### Reasons for and against offering patients letters and tapes

In the open-ended questions, oncologists, surgeons and GPs were asked to explain their views regarding specialists offering patients each of the letters or tape. Importantly, no single viewpoint can be generalized. Indeed, amongst each group of doctors – oncologists, surgeons and GPs – strong and divergent views were expressed.

### *Offering patients a copy of the letter written to the referring doctor/GP*

*Doctors in favour* of this practice argued on the grounds of both ethics and patient care. Their views were as follows:

- Patients have a right to this information, and it should be available to them irrespective of whether, or how much, they understand.
- Providing patients with a copy of this letter would be insurance from a medico-legal perspective.
- Open communication, demonstrated by offering patients a copy of this letter, is important in establishing trust.
- It gives patients a concise and clear record and often leads to more question-asking and clarification for both patient and doctor, facilitating management.
- It helps to clarify things for the patient's family and friends who get involved around the periphery.
- Patients value copies of such correspondence.

*Doctors opposed* to this practice expressed the following views:

- The letter to the referring doctor is personal correspondence and is not the patient's property. One surgeon expressed the

© Cancer Research Campaign 1999

view that if an oncologist intended to offer the patient a copy of this letter, the oncologist should first ask the referring doctor for their consent.
- The letter is written to inform the referring doctor, not the patient. Thus:
- Most patients would not understand the medical language used; this may cause increased and unnecessary confusion and anxiety.
- The 'clinical and cold' style of this letter is not appropriate for patients.
- The letter sometimes contains information that is confidential or may be detrimental to the patient; for example, criticism of previous management, blunt prognostic information and personal thoughts on sensitive psychosocial issues.
- If patients were to receive a copy of this letter, the information content may be altered/inhibited, and less frank.
- Providing patients with a copy of this letter reduces the feasibility of altering treatment plans with changes in circumstances.

### *Offering patients an individualized letter summarizing the consultation*

*Doctors in favour* of individualized letters expressed the following positive views:

- This letter could be tailored to the patient's individual needs/problems/concerns and would be more 'patient friendly'.
- Patients have difficulty taking information in at the consultation. Providing this letter may increase patient understanding and compliance.
- Patients would feel that their doctor is taking an individual interest in them, facilitating patient trust and confidence.
- One GP suggested this letter would be a useful back-up if patients return to them for a consultation before the 'doctor letter' arrived.

*Doctors opposed to* individualized letters expressed the following views:

- Providing patients with an individualized letter would be too time-consuming and too costly. As one doctor expressed it, 'There are only 24 hours in a day!'
- There is no guarantee that patients would not misunderstand or misinterpret the information conveyed in an individualized letter.
- Illness and circumstances change and therefore an individualized letter would be of minimal usefulness.
- If the medical jargon is excluded and the letter simplified there is a risk of it being perceived as paternalistic, or even concealing.
- Patients receive a lot of information and there is a problem of information overload.

### *Offering patients an audiotape recording of the cancer consultation*

*Doctors in favour* of consultation audiotapes expressed the following views:

- When offered, most patients want and appreciate this. One doctor said that he charged $2.00 to cover costs and most patients were willing to pay it.
- Audiotaping the consultation may provide effective medico-legal defence.

*Doctors opposed to* consultation audiotapes expressed the following views:

- Audiotaping the consultation is intrusive, inhibiting free-flowing and open discussion.
- Frequent interruptions during the consultation makes audio-taping cumbersome.
- Providing patients with an audiotape has no proven benefits and patients do not want them.
- Changes in the patient's condition makes this less useful.
- Medico-legally speaking, an audiotape recording of the consultation is 'risky'.
- Patients may have difficulty isolating the important points, or may focus on the wrong parts and ignore the real issues. Patients do not have the opportunity to clarify the information when reviewing the tape.
- Patient confidentiality may be compromised.
- An audiotape recording would miss the non-verbal components of the communication.

*Doctors in favour* of specialists offering patients these communication aids under certain conditions expressed the following views. Patients should be offered letters or audiotapes if:

- they request it; time and secretarial resources permit; they organize it or are willing to pay for it.
- they actively sought information during the consultation; they are worried, sceptical or unaccepting.
- their problem, or decision, is complicated.
- they are travelling or changing doctors.
- the initial shock of the illness has been dealt with; their prognosis is good; they are emotionally stable and coping well.
- all points in the letter have been discussed with the patient.
- they will be seen again soon so any questions can be answered; they have medical/paramedical knowledge; they are intelligent/well educated; the letter is succinct and easily understood.
- the referring doctor has given consent (professional etiquette).
- their family is happy for them to receive information.
- they speak English as a second language so they can review the information with an interpreter.
- audiotapes are useful if an individualized letter is not possible because of time constraints, or not useful because the patient is visually impaired or illiterate.
- the audiotape is especially helpful to patients who are in shock at their first consultation.

### Other interventions proposed by doctors

Oncologists, surgeons and GPs were asked if, in their opinion, there were any 'better' strategies than offering patients either a letter or tape to ensure patients are adequately/fully informed after a consultation. In response, 65% of oncologists, 58% of surgeons and 44% of GPs answered 'yes'. Each group of doctors was then asked to specify 'better' alternatives. Most doctors suggested a combination of the following 12 strategies:

### *Within consultation strategies*

1. Encourage patients to attend with a relative or friend.
2. During the consultation ask patients to explain what they

*© Cancer Research Campaign 1999*

understand about their situation and choices, and check to ensure that their information needs have been addressed.
3. Spend more time with patients, provide a clear explanation and repeat the important information.
4. Assure patients that there is no such thing as a 'dumb question'.

### *Information aids*

5. During the consultation make notes and illustrations for the patient to take with them.
6. Provide patients with general information booklets and cassettes, a videotape on the treatment proposed, and direct patients to appropriate sites on the internet.
7. Make scientific papers concerning proposed treatments available to patients.

### *Post-consultation follow-up*

8. Offer patients a repeat consultation to review the information and answer any questions arising.
9. Follow up each initial consultation with a phone call to clarify any information and answer any new questions. Also provide patients with a phone number by which they can contact you.
10. Advise patients to write down any questions they wish to ask next visit.
11. Utilize other members of the cancer care team to provide the patient with the opportunity to debrief.
12. Advise the patient to return to the GP to discuss their situation and options.

### DISCUSSION

Research investigating the effects of providing patients with a letter or audiotape of the cancer consultation has generated considerable support for incorporating these information aids into routine practice. The results of this study, however, indicate that oncologists and surgeons rarely offer patients either a consultation summary letter or an audiotape of the consultation. Few are in favour of offering these to all patients, although many doctors of each speciality indicated that they were in favour of offering these information aids in some circumstances. The majority of oncologists and surgeons, however, prefer different strategies, such as offering repeat consultations or spending more time with patients and assessing their understanding during the consultation.

The fact that less experienced (and presumably younger) surgeons were more likely to dictate letters to other doctors in front of patients and to favour the provision of consultation audiotapes, suggests that attitudes and practices may be changing. Perhaps this is in line with the shift towards a less paternalistic model of care and more collaborative decision-making. These were also significant predictors of attitudes in surgeons and, to a lesser extent, oncologists.

In relation to the three information aids – (1) a copy of the letter to the referring doctor, (2) an individualized letter, (3) a consultation audio tape – notably different opinions between medical disciplines were found. Amongst oncologists, it seems that offering patients a copy of the letter to the referring doctor is the least popular strategy, with 54% opposed, and an audiotape the most popular, with just 39% opposed. For surgeons and GPs, however, offering patients an audiotape recording was clearly the least preferred strategy with 66% and 73% opposed respectively. These differences may be attributable to experience. A considerably higher percentage of oncologists (21%) than surgeons (11%) provide consultation audiotapes in at least some cases.

The view expressed by many doctors that patients do not want such information aids is not supported by patient surveys. However, doctors from each medical discipline expressed other serious concerns about the provision of each information aid. Doctors were particularly concerned that the medical language used, and style of letters written to referring doctors/GPs, would cause increased confusion and anxiety for patients. Furthermore, doctors were concerned that consultants would be less frank, and limit the content of letters that were to be copied for patients.

Doctors opposed to offering patients individualized letters emphasized the time and cost in doing so, suggesting this option is impractical. In regard to consultation audiotapes, concerns focused on the potential negative effects of taping on the consultation interaction, and the possibility of patients misinterpreting aspects or missing the 'important' points. Doctors in favour of such aids emphasized their benefits of inducing greater trust and confidence in patients, and allowing better understanding, fuller discussion and increased involvement in decisions. Future research will need to assess the pros and cons of audiotaping consultations across a range of disciplines.

Divergent views on whether patients have a right to a copy of the letter to the referring doctor/GP and the potential medico-legal implications of each information aid remain matters for debate. Advice received by the authors from the Australian Medical Defence Organisations was that audiotapes would, in general, benefit the doctor in a medico-legal case.

The significant level of professional opposition to personalized information aids suggests that further research is required to establish the extent of consumer interest in, and demand for, such aids, and their benefits with regards to patient outcomes. It is important to acknowledge the professional objections raised and to explore ways of overcoming perceived barriers, if such information aids are to be incorporated more widely into routine clinical practice. Patient demand may in fact overtake these barriers; in a recent poll of attendees at a Colon Cancer Consensus meeting in Australia, only one in an audience of 200 doctors indicated that they would refuse permission sought by a patient to audiotape a consultation.

Given that the majority of doctors supported the provision to some or all patients of summary letters, and the majority of oncologists supported the provision of a consultation audiotape in at least some cases, it may be useful to establish a forum where physicians and patients can develop guidelines for the use of these information aids. Such guidelines might suggest appropriate criteria for determining when to offer such aids, as well as a suggested format. It may be appropriate, for example, to routinely offer patients a choice of communication aids that will cater to their individual information needs, rather than this decision falling on the doctor.

### ACKNOWLEDGEMENTS

This work was supported by the New South Wales Cancer Council.

### REFERENCES

Beisecker AE and Beisecker TD (1990) Patient information seeking behaviours when communicating with doctors. *Medical Care* **28**: 19–28

Blanchard CG, Labreque MS, Ruckdeschel JC and Blanchard EB (1988) Information and decision-making preferences of hospitalised cancer patients. *Soc Sci Med* **27**: 1139–1145

© Cancer Research Campaign 1999

Butow PN, Dunn SM, Tattersall MHN and Jones Q (1994) Patient participation in the cancer consultation: evaluation of a question prompt sheet. *Ann Oncol* **5**: 199–204

Butow PN, Maclean M, Dunn SM, Tattersall MHN and Boyer MJ (1997) The dynamics of change: cancer patients' preferences for information, involvement and support. *Ann Oncol* **8**: 857–863

Cassileth BR, Zupkis RV, Sutton-Smith K and March V (1980) Information and participation preferences among cancer patients. *Ann Intern Med* **92**: 832–836

Damian D and Tattersall MHN (1991) Letters to patients: improving communication in cancer care. *Lancet* **331**: 923–925

Derdiarian A (1987) Informational needs of cancer patients. A theoretical framework. Part 1. *Cancer Nursing* **10**: 107–115

Deutsch G (1992) Improving communication with oncology patients: taping the consultation. *J Clin Oncol* **4**: 46–47

Dunn SM, Butow PN and Tattersall MHN (1993) General information tapes inhibit recall. *J Clin Oncol* **11**: 2279–2286

Fallowfield L (1993) Giving sad and bad news. *Lancet* **341**: 476–478

Glaser BG and Strauss AL (1967) *The Discovery of Grounded Theory. Strategies for Qualitative Research*. Aldine Publishing: New York

Goldberg RJ (1984) Disclosure of information to adult cancer patients: issues and update. *J Clin Oncol* **2**: 948–954

Hack TF, Degner LF and Dyck DG (1994) Relationship between preferences for decisional control and illness information among women with breast cancer: a quantitative and qualitative analysis. *Soc Sci Med* **39**: 279–289

Hogbin B and Fallowfield L (1989) Getting it taped: the bad news consultation with cancer patients. *Br J Hosp Med* **41**: 330–333

Lancet (1995) Editorial: Who owns medical technology? *Lancet* **345**: 1125–1126

Lantos J (1993) Informed consent: the whole truth for patients? *Cancer* **72**: 2811–2815

Ley P (1988) *Communicating with Patients: Improving Communication, Satisfaction and Compliance*. Groom Helm: New York

Ley P and Spelman MS (1965) Communications in an out-patient setting. *Br J Soc Clin Psychol* **4**: 114–116

Mackillop WJ, Stewart WE, Ginsburg AD and Stewart SS (1988) Cancer patients' perceptions of their disease and its treatment. *Br J Cancer* **58**: 355–358

McHugh P, Lewis S, Ford S, Newkinds E, Rustin G, Coombes C, Smith D, O'Reilly S and Fallowfield L (1995) The efficacy of audiotapes in promoting psychological wellbeing in cancer patients: a randomised controlled trial. *Br J Cancer* **71**: 388–392

Simminoff LA, Fetting JH and Abeloff MD (1989) Doctor-patient communication about breast cancer adjuvant therapy. *J Clin Oncol* **7**: 1192–1200

Stockler M, Butow PN and Tattersall MHN (1993) The take home message: doctors' views on letters and tapes after a cancer consultation. *Ann Oncol* **4**: 549–552

Sutherland HJ, Llewellyn-Thomas HA, Lockwood GA and Tritchler DL (1989) Cancer patients: their desire for information and participation in treatment decisions. *J R Soc Med* **82**: 260–263

Tattersall MHN, Butow PN, Griffin A-M and Dunn SM (1994) The take-home message after a cancer consultation: a randomised trial of consultation audiotapes and individualised letters to patients. *J Clin Oncol* **12**: 1305–1311

Tattersall MHN, Butow PN and Ellis PM (1997) Meeting patients' information needs beyond the year 2000. *Support Care Cancer* **5**: 85–89

Turner S, Maher EJ, Young T and Vaughan Hudson G (1996) What are the information priorities for cancer patients involved in treatment decisions? An experienced surrogate study in Hodgkin's disease. *Br J Cancer* **73**: 222–227

Wiggers JH, O'Donovan K, Redman S and Sanson-Fisher R (1990) Cancer patient satisfaction with care. *Cancer* **66**: 610–616

*© Cancer Research Campaign 1999*
